# No Evidence of Association Between Soccer Heading and Cognitive Performance in Professional Soccer Players: Cross-Sectional Results

**DOI:** 10.3389/fneur.2019.00209

**Published:** 2019-03-12

**Authors:** Ana Carolina Rodrigues, Mariana Drummond Martins Lima, Leonardo Cruz de Souza, Celso Furtado, Cimar Eustáquio Marques, Lucas Gonçalves, Marcus Vinícius Lima, Rodrigo Pace Lasmar, Paulo Caramelli

**Affiliations:** ^1^Pró-Reitoria de Graduação, Reitoria da Universidade Federal de Minas Gerais, Belo Horizonte, Brazil; ^2^Grupo de Pesquisa em Neurologia Cognitiva e do Comportamento, Departamento de Clínica Médica, Faculdade de Medicina da Universidade Federal de Minas Gerais, Belo Horizonte, Brazil; ^3^Departamento Médico do América Futebol Clube, Belo Horizonte, Brazil; ^4^Departamento de Análise de Desempenho do Clube Atlético Mineiro, Belo Horizonte, Brazil; ^5^Departamento Médico do Clube Atlético Mineiro, Belo Horizonte, Brazil; ^6^Faculdade de Ciências Médicas de Minas Gerais, Belo Horizonte, Brazil

**Keywords:** soccer, heading, injury, brain, cognitive functioning

## Abstract

Although the scientific community has focused on the effects of concussions in contact sports, the role of subconcussive impacts, as it can occur during soccer heading, has recently gained attention, considering that it may represent an additional mechanism of cumulative brain injury. The aim of this study is to investigate the effects of soccer heading on cognitive functioning in active professional soccer players. Male soccer players (*n* = 44), from two soccer teams that play in the Brazilian A Series Championship, and non-athletes (*n* = 47), comparable in age and education, were submitted to cognitive assessment, consisting of computerized and conventional neuropsychological testing (Neupsilin battery). In the computerized cognitive assessment, soccer players performed better than controls on reaction time measures in general motor coordination, executive functioning and memory tests, and on accuracy measures in executive functioning tests. There were no significant differences between groups on the Neupsilin battery. A comparison between two sub-groups of soccer players, based on the self-reported number of headings, did not show significant differences on tests performance. No significant correlations were found between an estimate of exposure to heading during professional soccer career and cognitive performance. Our data demonstrate no evidence of cognitive impairment in soccer players, compared to non-athletes, and no association between heading exposure and performance on neuropsychological tests. Longitudinal investigations, including neuroimaging assessment, will help to clarify whether soccer heading may be associated with brain injury and cognitive dysfunction.

## Introduction

Soccer is the most popular sport in the world and is unique, in comparison to other sports, as it is the only sport in which participants purposely use their head to hit the ball. Heading is considered an offensive or defensive move whereby the player's unprotected head is used to deliberately impact the ball and direct it during play. A soccer player can be subjected to an average of 6–12 headings per competitive game, where the ball reaches high velocities (1). Moreover, heading training is common in practice sessions. Although the scientific community has focused on the effects of concussion in contact sports, the possible consequences of subconcussive impacts, as it can occur during heading, have recently gained attention. The term “subconcussive” was proposed to describe the impact to the head that may cause neuronal dysfunction in the absence of concussive symptoms (2). Since repetitive subconcussive impacts are substantial in soccer, their clinical significance as a potential cause of traumatic brain injury and a risk of later development of neurodegenerative disease may represent a public health problem.

Research on the effects of soccer heading on brain structure and function has produced mixed results (3). A small number of studies have investigated the impact of heading on brain structure by using neuroimaging techniques. While some of these studies have found evidence of abnormalities (4–6), others have failed to show any evidence of brain damage in soccer players (7–9). Also, another line of research has specifically investigated biochemical markers of brain injury and their relationship with head impacts in soccer players. However, while some authors have demonstrated associations between heading and biochemical signs of brain damage (10–15), others have not corroborated these findings (16, 17).

Similarly to the research addressing the effects of soccer heading on brain structure, investigations on the effects of subconcussive impacts on brain function has produced unclear results. A recent study demonstrated an association between soccer heading and immediate and transient changes in electrophysiological measures of brain function (18). There has been a growing number of studies focusing on cognitive functioning in soccer players, but their results are also ambiguous, with some suggesting an association between heading practice and cognitive impairment (5, 6, 18–27) and others finding no evidence of association (8, 9, 15, 28–36). However, it is important to emphasize that these studies vary considerably in sample size, age of subjects, soccer practice level, estimation of heading frequency, cognitive assessment methods, presence and composition of control groups, and study design.

Only two cross-sectional studies conducted cognitive assessment of soccer groups composed entirely by active professional soccer players, with contradictory results. An investigation conducted by Matser et al. (20) compared male professional soccer players with a group of elite non-contact sport athletes, by using an extensive neuropsychological test battery, and showed poorer performance on verbal and visual memory, planning, and visuoperceptual processing tasks in the soccer group. Moreover, an increasing number of headings incurred during soccer participation was associated negatively with cognitive functioning. Field position also influenced performance on neuropsychological testing, as forward and defensive players performed significantly poorer than midfield players and goalkeepers on some tasks. On the other hand, a study conducted by Straume-Naesheim et al. (32) examined performance of male professional soccer players on computerized neuropsychological tests, measuring motor function, decision-making, simple, divided and complex attention, working memory, and learning and memory, and showed no association between estimated match or lifetime heading exposure and cognitive performance. Only 1.5% of the players qualified as outliers for one or more subtasks when compared with the normal range.

As described above, the research about the effects of heading on brain has produced intriguing results, but the findings are still inconclusive. It has been estimated that professional soccer players head the ball more than 2,000 times during their careers (28). However, as mentioned, few studies focused on active professional players (7, 8, 11–14, 20, 32), and only one involved computerized cognitive assessment (32). To our knowledge, there is currently no study about the possible deleterious effects of heading on brain functioning involving concomitantly a sample of active professional soccer players and a combination of computerized and conventional cognitive assessment.

Also, only two studies (20, 32) evaluating active professional players mentioned the educational level of the participants, a variable that can significantly influence cognitive performance (37). Furthermore, it is noteworthy the absence of research about the effects of soccer heading on brain in developing countries, as is the case of Brazil, where the schooling level of the population tends to be lower when compared to developed countries. Given the role of education in improving cognitive reserve (38), it is particularly important to investigate individuals who, besides being subjected to repetitive head impacts in their professional activity, may be more cognitively vulnerable, due to low educational level.

In this cross-sectional study, we aimed to investigate the effects of soccer heading on cognitive functioning, by comparing the performance of active professional soccer players and control subjects on computerized and conventional neuropsychological tests.

## Materials and Methods

### Participants

The study sample consisted of two groups of male participants: 44 active professional soccer players (mean age = 24.6 ± 4.5 years; mean educational level = 10.5 ± 1.7 years), from two soccer teams that play in the Brazilian A Series Championship, based in Belo Horizonte, Brazil—*América Futebol Clube* and *Clube Atlético Mineiro*—and 47 non-athletes control subjects (mean age = 25.9 ± 4.2 years; mean educational level = 11.0 ± 1.5 years). The groups were comparable in terms of age [*t*_(89)_ = −1.38; *p* = 0.171] and education (U = 846.00; *p* = 0.112). The group of soccer players consisted of nine forwards, 14 midfielders, 15 defenders, and 6 goalkeepers. We decided not to exclude any playing position, in order to investigate a wide range of exposure to headings among soccer players. All athletes perform ~15 h of training sessions per week and usually play one or, during most of the soccer season, two competitive matches weekly. The control group was composed by non-athletes, who work as doormen or guards and do not practice soccer, or practice it occasionally and at a recreational level. All subjects provided written informed consent to participate in the study, which was approved by the Ethics Committee of the Federal University of Minas Gerais.

### Clinical Information

We applied a sociodemographic questionnaire, to characterize each subject, and parts of the Mini International Neuropsychiatric Interview (39), to investigate possible psychiatric disorders. Nine soccer players (20.4%) and four controls (8.5%) reported a lifetime history of concussion, but there was no difference in rate of concussed subjects between groups [$X_{\left(1\right)}^{2}$ = 2.64; *p* = 0.104]. None of the participants reported using drugs with effects on central nervous system, nor exhibited major depressive episode or alcohol dependence.

### Heading Exposure

The self-reported number of headings per game among soccer players ranged from 0 to 22.5 (mean = 7.5 ± 5.8 headings), with median value of 5.75 headings, which allowed us to analyze two sub-groups of players (below and above the median number of headings per game), comparable in terms of age [*t*_(42)_ = 0.72; *p* = 0.471], education [*t*_(42)_ = −0.78; *p* = 0.437] and rate of concussion [$X_{\left(1\right)}^{2}$ = 1.25; *p* = 0.262]. The number of competitive matches played by each athlete during his professional soccer career until 2016, obtained from a sport database, varied from 0 to 517 (mean = 98.8 ± 127.4 competitive matches). The values of these two variables were then multiplied in order to estimate the exposure to heading during professional soccer career of each player. The estimated total number of headings ranged from 0 to 6105 (mean = 601.5 ± 1056.7 headings). Since the self-report on the number of headings per game is not an accurate measure and may not correspond to the actual values, we objectively counted the number of headings for each player in some competitive matches. The number of headings performed by 16 athletes in 42 games was directly measured by an observer. Considering this sub-sample of players, a comparison between the self-reported and the actual number of headings per game revealed no significant difference [*t*_(30)_ = 0.00; *p* = 1.000]. Also, a significant correlation was found between the self-reported and the actual values (Pearson correlation coefficient = 0.777; *p* = 0.000).

### Cognitive Assessment

The first part of the cognitive assessment consisted of computerized neuropsychological tests, measuring general motor coordination, memory, attention and executive functioning. All tests were designed on the E-Prime software (40), which has been used for investigations of many cognitive functions [e.g., (41)]. The participants accomplished the tasks with the preferred hand, and the answers were registered on the computer keyboard. In all tests, reaction time was measured in milliseconds (ms) and accuracy was measured as the percentage of correct responses. The tests were preceded by standardized instructions, which included answering as quickly as possible, as well as some practice trials, except in the memory tests. This assessment lasted approximately 35 min (min). Below, we present a brief description of the computerized tests, applied in the same order for all subjects.

#### Simple Reaction Time Test

In this test, which measures general motor coordination, the subject is asked to press the “1” key in response to the symbol “^*^,” which is flashed on the center of the screen at varying time intervals, ranging from 0.5 to 2.5 seconds (s). The test contains 40 trials and a single block.

#### Immediate Memory Test

In this test, which measures immediate verbal memory, 20 words are sequentially presented on the center of the screen for memorization. The list includes two words from each of the following categories—animal, body, clothing, device, food, furniture, nature, object, place, and transport—and each word remains on the screen for 2 s. Subsequently, sets composed by four words, numbered 1–4, are exhibited, with each set containing only one word previously presented in the list. For each set, the subject is asked to respond which word was shown in the list of 20 words, by pressing the “1,” “2,” “3,” or “4” keys. The test contains three blocks of 20 trials, each one presenting the same 20 words for memorization, but in a different display order. Reaction time and accuracy are calculated as the average values from the three blocks.

#### Attention Test

This test, which measures visual selective attention, consists of the presentation of sets composed by five figures, sequentially displayed, each one containing a target figure on the upper part of the screen and, below it, a group of four figures similar to one another and to the target figure. The subject is required to indicate whether the target figure is or is not present in the group below it, by pressing the “Yes” or “No” keys, respectively. The test contains 60 trials and a single block.

#### Executive Functioning Tests

These tests assess the three core components of executive functioning—task switching, working memory, and response inhibition, respectively by the Number-letter test, the Two-back test, and the Stroop test.

##### Number-letter-test

In this test, adapted from Miyake et al. (42), a number-letter pair is presented in one of four quadrants on the screen. The subject is instructed to indicate whether the number is odd or even (2, 4, 6, and 8 for even; 3, 5, 7, and 9 for odd) when the number-letter pair is presented in the top two quadrants, by pressing the “Even” or “Odd” keys, and whether the letter is a consonant or a vowel (G, K, M, and R for consonant; A, E, I, U for vowel) when the number-letter pair is presented in the bottom two quadrants, by pressing the “Consonant” or “Vowel” keys. The number-letter pair is exhibited only in the top two quadrants in the first block, only in the bottom two quadrants in the second block, and randomly around all four quadrants in the third block. Thus, only the third block requires subjects to shift between the two types of categorization operations. The test contains 32 trials in the first two blocks and 64 trials in the third block. Reaction time and accuracy are calculated as the difference between the values from the third block and the average values from the first two blocks, which means the cost of task switching.

##### Two-back-test

This test, adapted from Zheng et al. (43), consists of the presentation of quickly changing numbers, from 1 through 9, on the center of the screen. The subject is asked to monitor the sequence of numbers, by pressing the “Enter” key whenever a stimulus is the same as the one presented two trials before, which requires working memory. The target numbers account for 25% of the total amount of numbers. The test contains 160 trials and a single block.

##### Stroop-test

In this test, adapted from Zheng et al. (43), the words “Green,” Blue,” “Red,” and “Yellow” are sequentially displayed on the center of the screen in one of two possible conditions: congruence, when the meaning of the word matches the ink color (e.g., the word “Red” written in red), and incongruence, when the meaning of the word differs from the ink color (e.g., the word “Red” written in blue). Each condition accounts for 50% of the total amount of words. The subject is required to name the color in which each word is written, refraining from reading the word, by pressing the “Green,” Blue,” “Red,” and “Yellow” keys. The test contains 72 trials and a single block. Reaction time and accuracy are calculated as the difference between the values from the incongruent condition and those from the congruent condition, which means the cost of response inhibition.

#### Delayed Memory Test

In this test, which is applied ~30 min after the immediate memory test and measures delayed verbal memory, 40 words, from which 50% were displayed previously, are sequentially presented on the center of the screen. The subject is instructed to respond whether each word was or was not present in the list of 20 words exhibited in the first memory test, by pressing the “Yes” or “No” keys. The test contains 40 trials and a single block.

The second part of the cognitive assessment, applied immediately after the first part, involved the Brief Neuropsychological Assessment Battery Neupsilin (44), a previously validated instrument for use in the Brazilian population (45), which briefly examines the neuropsychological profile of both clinical and healthy populations. The battery, composed by 32 short tasks, evaluates time and spatial orientation, attention, perception, memory, arithmetic abilities, language, praxis, problem solving, and verbal fluency. Overall, the tasks involve vocal, written or sign responses, and each one receives a specific score, referred as the measured variable. The tasks were preceded by standardized instructions and this assessment lasted ~35 min.

### Statistical Analysis

After testing the normality of each variable, utilizing the Kolmogorov-Smirnov test, mean comparisons between groups were performed using Student's *t*-test for independent samples for variables with normal distribution and the Mann-Whitney *U*-test for non-normally distributed variables. Correlations between the performance of soccer players in tests and estimate of exposure to heading during professional soccer career were calculated using Pearson's correlation test, controlling for lifetime history of concussion. Categorical variables were compared using the Chi-square test. The significance level was set to 5% (*p* < 0.05) for all tests. All statistical analyses were performed with SPSS (Version 19.0. IBM Corp).

## Results

Data comparing performance of soccer players and control subjects are shown in Tables 1–3. On computerized cognitive assessment (Table 1), soccer players performed better on reaction time measures in the simple reaction time test [*t*_(89)_ = −2.91; *p* = 0.004], the immediate memory test [*t*_(89)_ = −2.01; *p* = 0.048], the number-letter test [*t*_(89)_ = −3.35; *p* = 0.001], the two-back test [*t*_(89)_ = −2.05; *p* = 0.043], and the delayed memory test [*t*_(89)_ = −2.33; *p* = 0.022]. Moreover, soccer players performed better on accuracy measures in the number-letter test (U = 747.50; *p* = 0.022) and in the two-back test [*t*_(89)_ = 3.77; *p* = 0.000]. The performance in the other variables of the computerized neuropsychological tests was similar between groups.

**Table 1:** Comparison between soccer players and control subjects on computerized cognitive assessment.

		**Soccer players (*****n*** **= 44)**		**Control subjects (*****n*** **= 47)**		***p***
**Test**	**Variable**	**Mean**	**Standard deviation**	**Mean**	**Standard deviation**	
Simple reaction time	Reaction time	294	31	316	41	**0.004**
	Accuracy	100.00	0.00	99.97	0.18	0.333*
Attention	Reaction time	2448	726	2702	624	0.077
	Accuracy	93.67	5.71	94.53	4.73	0.418*
Immediate memory^a^	Reaction time	2599	699	2919	810	**0.048**
	Accuracy	88.71	7.70	86.77	11.16	0.341
Number-letter^a^	Reaction time	539	148	662	194	**0.001**
	Accuracy	−0.85	3.11	−3.39	5.52	0.022*
Two-back	Reaction time	525	90	566	97	**0.043**
	Accuracy	97.71	2.65	94.54	4.92	**0.000**
Stroop^a^	Reaction time	166	101	159	141	0.785
	Accuracy	−1.64	3.19	−2.83	5.83	0.883*
Delayed memory	Reaction time	929	190	1030	220	**0.022**
	Accuracy	93.92	5.12	91.80	7.79	0.128

The p-values refer to the Student's t-test for independent samples, except those marked with

* which refer to the Mann-Whitney U-test. The black p-values indicate significant differences at a 5% level. Reaction time is shown in milliseconds, and accuracy is shown in the percentage of correct answers.

a *In the Immediate memory test, the Number-letter test and the Stroop test, reaction time and accuracy are calculated as described in the Materials and Methods section*.

With regard to the Neupsilin battery (Table 2), there were no significant differences between soccer players and control subjects. As presented in Table 3, a comparison involving the rate of subjects with deficit scores, i.e., values below normative cut-off points for age- and education-matched population, which are calculated by subtracting 1.5 standard deviation from the mean values, also did not reveal any difference between groups.

**Table 2:** Comparison between soccer players and control subjects on Neupsilin battery.

		**Soccer players (*****n*** **= 44)**		**Control subjects (*****n*** **= 47)**		***p***
**Test**	**Total score**	**Mean**	**Standard deviation**	**Mean**	**Standard deviation**	
Time and spatial orientation	8	7.86	0.50	7.97	0.14	0.145*
Attention	27	23.56	2.90	23.08	2.08	0.362
Perception	12	11.13	0.95	11.19	0.87	0.877*
Memory	84	59.13	7.16	56.12	8.20	0.066
Arithmetic abilities	8	6.88	1.60	6.38	1.84	0.199*
Language	53	50.31	1.69	50.06	2.38	0.557
Praxis	22	18.15	2.01	17.34	2.39	0.082
Problem solving	2	1.68	0.56	1.55	0.50	0.132*
Verbal fluency	11	5.18	1.46	5.12	1.24	0.836*

The p-values refer to the Student's t-test for independent samples, except those marked with

* *which refer to the Mann-Whitney U-test. The measured variables are scores for each test*.

**Table 3:** Comparison between soccer players and control subjects with regard to the rate of subjects with deficit scores on Neupsilin battery.

**Test**	**Soccer players (*n* = 44) (%)**	**Control subjects (*n* = 47) (%)**	***p***
Time and spatial orientation	9.09	2.12	0.145
Attention	2.27	0.00	0.299
Perception	6.81	4.25	0.592
Memory	2.27	8.51	0.192
Arithmetic abilities	25.00	42.55	0.077
Language	47.72	46.80	0.930
Praxis	11.36	17.02	0.441
Problem solving	25.00	42.55	0.077
Verbal fluency	13.63	10.63	0.661

*The p-values refer to the chi-square test. Deficit scores are values below normative cut-off points for age- and education-matched population, which are calculated by subtracting 1.5 standard deviation from the mean values*.

A comparison between two sub-groups of soccer players, separated by the median value of the self-reported number of headings per game, did not show significant differences on test results (Tables 4,5).

**Table 4:** Comparison between sub-groups of soccer players on computerized cognitive assessment.

		**Soccer players who perform >5.75 headings per game (*****n*** **= 22)**		**Soccer players who perform < 5.75 headings per game (*****n*** **= 22)**		***p***
**Test**	**Variable**	**Mean**	**Standard deviation**	**Mean**	**Standard deviation**	
Simple reaction time	Reaction time	297	33	290	29	0.478
	Accuracy	100.00	0.00	100.00	0.00	–
Attention	Reaction time	2240	503	2657	858	0.056
	Accuracy	93.25	7.17	94.08	3.87	0.634
Immediate memory^a^	Reaction time	2544	722	2655	687	0.607
	Accuracy	87.80	7.26	89.62	8.19	0.440
Number-letter^a^	Reaction time	548	152	530	147	0.697
	Accuracy	−1.63	3.14	−0.07	2.95	0.096
Two-back	Reaction time	527	94	523	87	0.894
	Accuracy	97.12	3.27	98.29	1.73	0.150
Stroop^a^	Reaction time	169	107	163	97	0.833
	Accuracy	−1.13	3.17	−2.14	3.20	0.299
Delayed memory	Reaction time	915	197	943	186	0.640
	Accuracy	93.75	4.92	94.09	5.43	0.828

*The p-values refer to the Student's t-test for independent samples. Reaction time is shown in milliseconds, and accuracy is shown in the percentage of correct answers*.

a *In the Immediate memory test, the Number-letter test and the Stroop test, reaction time and accuracy are calculated as described in the Materials and methods section*.

**Table 5:** Comparison between sub-groups of soccer players on Neupsilin battery.

		**Soccer players who perform >5.75 headings per game (*****n*** **= 22)**		**Soccer players who perform < 5.75 headings per game (*****n*** **= 22)**		***p***
**Test**	**Total score**	**Mean**	**Standard deviation**	**Mean**	**Standard deviation**	
Time and spatial orientation	8	7.81	0.66	7.90	0.29	0.962*
Attention	27	22.90	3.16	24.22	2.52	0.134
Perception	12	11.13	0.94	11.13	0.99	1.000
Memory	84	57.09	6.17	60.18	8.03	0.339
Arithmetic abilities	8	6.86	1.85	6.90	1.34	0.926
Language	53	50.31	1.61	50.31	1.80	1.000
Praxis	22	17.81	2.19	18.50	1.79	0.266
Problem solving	2	1.72	0.55	1.63	0.58	0.526*
Verbal fluency	11	5.18	1.40	5.18	1.56	1.000

The p-values refer to the Student's t-test for independent samples, except those marked with

* *which refer to the Mann-Whitney U-test. The measured variables are scores for each test*.

As shown in Tables 6, 7, no significant correlations were found between estimate of exposure to heading during professional soccer career and cognitive performance.

**Table 6:** Correlations between performance of soccer players on computerized cognitive assessment and estimate of exposure to heading during professional soccer career.

		**Estimate of exposure to heading during professional soccer career**	
**Test**	**Variable**	**Correlation coefficient (*r*)**	***p***
Simple reaction time^a^	Reaction time	0.13	0.386
	Accuracy	–	–
Attention	Reaction time	−0.11	0.475
	Accuracy	0.15	0.315
Immediate memory^b^	Reaction time	−0.00	0.989
	Accuracy	0.19	0.203
Number-letter^b^	Reaction time	0.05	0.727
	Accuracy	0.14	0.369
Two-back	Reaction time	0.00	0.998
	Accuracy	0.02	0.897
Stroop^b^	Reaction time	−0.26	0.084
	Accuracy	0.10	0.504
Delayed memory	Reaction time	0.25	0.098
	Accuracy	0.21	0.162

The correlations were measured by Pearson's correlation test, controlling for the variable “lifetime history of concussion.”

a *The correlation test was not performed for accuracy in the simple reaction time test since all subjects had 100% of correct responses*.

b *In the Immediate memory test, the Number-letter test and the Stroop test, reaction time and accuracy are calculated as described in the Materials and methods section*.

**Table 7:** Correlations between performance of soccer players on Neupsilin battery and estimate of exposure to heading during professional soccer career.

	**Estimate of exposure to heading during professional soccer career**	
**Test**	**Correlation coefficient (*r*)**	***p***
Time and spatial orientation	0.01	0.934
Attention	0.06	0.665
Perception	0.13	0.403
Memory	−0.10	0.521
Arithmetic abilities	0.01	0.949
Language	0.26	0.084
Praxis	−0.00	0.954
Problem solving	0.02	0.887
Verbal fluency	0.24	0.118

The correlations were measured by Pearson's correlation test, controlling for the variable “lifetime history of concussion.”

Moreover, as presented in Tables 8, 9, cognitive performance was similar between soccer players with and without history of concussion, comparable in terms of age [*t*_(9.63)_ = 1.36; *p* = 0.204] and education (*U* = 122.50; *p* = 0.282), except in the perception test of Neupsilin battery, with athletes with a lifetime history of concussion showing a slightly lower score (*U* = 89.00; *p* = 0.032).

**Table 8:** Comparison between soccer players with and without history of concussion on computerized cognitive assessment.

		**Soccer players with history of concussion (*****n*** **= 9)**		**Soccer players without history of concussion (*****n*** **= 35)**		***p***
**Test**	**Variable**	**Mean**	**Standard deviation**	**Mean**	**Standard deviation**	
Simple reaction time	Reaction time	294	30	294	31	0.969
	Accuracy	100.00	0.00	100.00	0.00	–
Attention	Reaction time	2561	798	2419	716	0.607
	Accuracy	95.92	2.90	93.09	6.13	0.188
Immediate memory^a^	Reaction time	2588	788	2602	687	0.956
	Accuracy	88.88	7.81	88.66	7.79	0.939
Number-letter^a^	Reaction time	484	165	553	143	0.216
	Accuracy	−1.21	1.87	−0.76	3.37	0.700
Two-back	Reaction time	505	77	530	93	0.459
	Accuracy	97.77	2.91	97.69	2.62	0.936
Stroop^a^	Reaction time	145	114	171	98	0.504
	Accuracy	−0.61	1.85	−1.90	3.42	0.141
Delayed memory	Reaction time	905	179	935	194	0.683
	Accuracy	91.11	6.74	94.64	4.46	0.168

The p-values refer to the Student's t-test for independent samples. Reaction time is shown in milliseconds, and accuracy is shown in the percentage of correct answers.

a *In the Immediate memory test, the Number-letter test and the Stroop test, reaction time and accuracy are calculated as described in the Materials and methods section*.

**Table 9:** Comparison between soccer players with and without history of concussion on Neupsilin battery.

		**Soccer players with history of concussion (*****n*** **= 9)**		**Soccer players without history of concussion (*****n*** **= 35)**		***p***
**Test**	**Total score**	**Mean**	**Standard deviation**	**Mean**	**Standard deviation**	
Time and spatial orientation	8	8.00	0.00	7.82	0.56	0.293*
Attention	27	24.88	2.14	23.22	3.00	0.128
Perception	12	10.55	1.01	11.28	0.89	0.032*
Memory	84	63.22	6.33	58.08	7.06	0.054
Arithmetic abilities	8	7.11	1.05	6.82	1.72	0.938*
Language	53	50.44	1.81	50.28	1.69	0.805
Praxis	22	18.77	1.56	18.00	2.10	0.306
Problem solving	2	1.66	0.50	1.68	0.58	0.736*
Verbal fluency	11	5.22	1.48	5.17	1.48	0.927

The p-values refer to the Student's t-test for independent samples, except those marked with

* *which refer to the Mann-Whitney U-test. The black p-value indicates significant difference at a 5% level. The measured variables are scores for each test*.

## Discussion

To our knowledge, this is the first study investigating the effects of soccer heading on cognitive functioning involving concomitantly a sample of active professional soccer players and a combination of computerized and conventional cognitive assessment. Our data demonstrated better performance of soccer players, compared to controls, on computerized neuropsychological tests, namely reaction time measures in general motor coordination, executive functioning and memory tests, and accuracy measures in executive functioning tests. No significant difference between groups emerged on Neupsilin battery.

Interestingly, from the seven variables showing significant differences between groups on computerized cognitive assessment, five variables refer to reaction time and only two refer to accuracy. The advantage of soccer players on reaction time measures, when compared to controls, could be at least partially explained by better general motor coordination, since athletes had shorter reaction times also in the simple reaction time test. There is evidence that participation in sports relates to faster processing speed on measures of simple reaction time (46). The better performance of soccer players on accuracy measures in executive functioning tests, specifically assessing task switching and working memory, may be associated to team sport practice, which demands strategic decision-making in complex and quickly changing contexts. Previous research, although not focusing on the effects of soccer heading on brain function, has demonstrated better executive functioning in soccer players in comparison to general population (47).

It is also noteworthy that while computerized cognitive assessment revealed some differences between soccer players and controls, conventional neuropsychological tests did not show any difference between groups. This is in line with studies investigating the reliability of computerized cognitive assessment, which have suggested that measures of response speed are more sensitive in detecting neuropsychological changes than measures of response accuracy in healthy young adults (48).

Considering the literature investigating the effects of soccer heading on cognition (5, 6, 8, 9, 15, 18–36), as pointed out earlier, only two cross-sectional studies involved cognitive assessment exclusively of active professional soccer players. One study (20) demonstrated an association between soccer heading and cognitive impairment, but the other (32) did not corroborate this finding. However, it is important to emphasize a distinction between these two studies with regard to the control groups. While Matser et al. (20) included elite noncontact sport athletes as controls, Straume-Naesheim et al. (32) compared the performance of soccer players with normative values for the general population. Taking into account the evidence of positive effects of physical exercise practice on cognition (49), a comparison involving a control group composed by non-athletes, as is also the case of our study, could difficult to some extent the detection of possible deficits on cognitive performance of soccer players when compared to controls. Moreover, another relevant observation is that our control group was composed by doormen and guards, professions that do not normally involve high cognitive demand, which may also have influenced the results.

A limitation of our study refers to the composition of the control group, which involved some subjects who practiced soccer, albeit at recreational level. It was not possible to measure the amount of irregular exposure to headings within the control group. However, it is important to emphasize that soccer is an extremely popular sport in Brazil, among all age ranges and socioeconomic conditions. Therefore, it would not be surprising that the control group involved subjects who practiced soccer as a leisure activity, since that is part of the country's culture. We do not believe that such a limitation has significantly affected our results, considering the difference in sport practice level between professional soccer players and non-athletes who play soccer recreationally.

There were no significant differences on test variables between soccer players who reported larger or smaller number of headings per game. Similarly, no significant correlations were found between estimate of exposure to heading during professional soccer career and cognitive performance, in contrast with the study by Matser et al. (20) and in accordance with the results by Straume-Naesheim et al. (32). However, another important distinction between these two studies refers to the estimate of heading exposure. In the first study, the authors estimated the total number of headings in only one season, whereas in the second one, as in our study, the estimate involved the entire professional soccer career. In any case, some considerations must be taken with respect to the estimate of exposure to heading. In most studies, as in our investigation, such an estimate is based on self-reported information provided by soccer players, a method with unknown validity. The self-report on the number of headings may be quite variable and potentially biased, which may explain the high standard deviation of the variable regarding long-term heading exposure. In addition, even assuming a correspondence between the self-reported and the actual values, the number of headings may, in fact, vary across matches and sport seasons, an aspect that was not addressed in our estimate. Moreover, when estimating the exposure to heading during professional soccer career, we considered only the number of competitive matches played by each athlete, excluding the training sessions over time, which may considerably underestimate heading exposure. Also, heading technique may vary among soccer players (5), resulting in situations of greater or lesser risk for head injury, a variable that was not controlled for in our study. Even though few soccer players in our sample reported a lifetime history of concussion, the results showed a relative homogeneity of cognitive performance in the soccer group, since only one test variable showed a significant difference between athletes with and without history of concussion.

Although no evidence of brain function impairment in soccer players has been found, we cannot draw conclusions about the issue involving soccer heading and brain injury from cross-sectional data. Moreover, the selection of soccer players from only two soccer teams may also limit the generalizability of the findings. Further longitudinal studies involving larger samples will be crucial for better understanding of this matter, which remains controversial. The first longitudinal study assessing brain structure and function in active professional soccer players did not show evidence of abnormalities or deficits over a period of 5 years (8), but these results need corroboration. It could be argued that soccer heading does not involve enough force to represent a subconcussive impact and, therefore, does not affect cognitive function. In order to investigate this hypothesis, future research should also combine heading exposure, cognitive and biomechanical data, and their potential relationships. As emphasized by some authors (50), although a spectrum of chronic neurological injuries associated with sports-related concussive and subconcussive brain trauma may occur, a primary concern involves chronic traumatic encephalopathy, which has already been reported in studies involving soccer players (51, 52), as well as other contact sport athletes (53, 54). The prevalence of chronic traumatic encephalopathy is still unknown and *in vivo* diagnosis is not currently possible, which may enhance the concern about potential consequences of soccer heading.

In summary, our data demonstrated no evidence of cognitive dysfunction in active professional soccer players compared to non-athletes. Conversely, soccer players even outperformed control subjects on some computerized neuropsychological tests. Among soccer players, there were no significant differences between those who reported larger or smaller number of headings per game and no significant correlations between estimate of exposure to heading during professional soccer career and cognitive performance. Longitudinal investigations will help to clarify whether heading may be associated with brain injury and cognitive dysfunction.

## Data Availability

The datasets generated for this study are available on request to the corresponding author.

## Ethics Statement

This study was carried out in accordance with the recommendations of the Ethics Committee of the Federal University of Minas Gerais, with written informed consent from all subjects. All subjects gave written informed consent in accordance with the Declaration of Helsinki. The protocol was approved by the Ethics Committee of the Federal University of Minas Gerais.

## Author Contributions

AR and PC are responsible for the conception and design of the study. AR was responsible for cognitive assessment. MDL, CF, CM, MVL, and RL contributed to data collection. LG counted the number of headings per player in competitive matches. AR and MDL performed the statistical analysis. AR drafted the manuscript. LdS and PC revised it critically. All authors read and approved the submitted version.

### Conflict of Interest Statement

The authors declare that the research was conducted in the absence of any commercial or financial relationships that could be construed as a potential conflict of interest.
